# Potential Therapeutic Strategies for Hypertension-Exacerbated Cardiotoxicity of Anticancer Drugs

**DOI:** 10.1155/2016/8139861

**Published:** 2016-10-17

**Authors:** Robin K. Kuriakose, Rakesh C. Kukreja, Lei Xi

**Affiliations:** ^1^Pauley Heart Center, Division of Cardiology, Department of Internal Medicine, Virginia Commonwealth University, Richmond, VA 23298-0204, USA; ^2^School of Sports Medicine and Health, Chengdu Sport University, Chengdu 610041, China

## Abstract

Despite their recognized cardiotoxic effects, anthracyclines remain an essential component in many anticancer regimens due to their superior antitumor efficacy. Epidemiologic data revealed that about one-third of cancer patients have hypertension, which is the most common comorbidity in cancer registries. The purpose of this review is to assess whether anthracycline chemotherapy exacerbates cardiotoxicity in patients with hypertension. A link between hypertension comorbidity and anthracycline-induced cardiotoxicity (AIC) was first suggested in 1979. Subsequent preclinical and clinical studies have supported the notion that hypertension is a major risk factor for AIC, along with the cumulative anthracycline dosage. There are several common or overlapping pathological mechanisms in AIC and hypertension, such as oxidative stress. Current evidence supports the utility of cardioprotective modalities as adjunct treatment prior to and during anthracycline chemotherapy. Several promising cardioprotective approaches against AIC pathologies include dexrazoxane, early hypertension management, and dietary supplementation of nitrate with beetroot juice or other medicinal botanical derivatives (e.g., visnagin and Danshen), which have both antihypertensive and anti-AIC properties. Future research is warranted to further elucidate the mechanisms of hypertension and AIC comorbidity and to conduct well-controlled clinical trials for identifying effective clinical strategies to improve long-term prognoses in this subgroup of cancer patients.

## 1. Introduction

The incidence of comorbidities such as hypertension and malignancy increases with the growing population of the elderly in the major industrial countries [1]. One study showed that the rate of hypertension comorbidity in cancer patients was around 30% [2]. In order to provide optimal treatment to this subpopulation of cancer patients with hypertension comorbidity, it is necessary to understand the potential effects of hypertension and anthracycline treatment on short- and long-term cardiac function.

Current chemotherapy regimens are typically administered in combination with other drugs, rather than monotherapy, to maximize the efficacy of anticancer treatment. Regarding the most widely used anticancer chemotherapeutic agents, their toxic effects on organs in the cardiovascular system have drawn considerable attention during the past few decades. While anthracyclines are the most well-known and extensively investigated drugs with severe cardiotoxic effects, other drugs such as cyclophosphamide, a component of CHOP therapy (i.e., C: Cytoxan®, cyclophosphamide; H: Adriamycin®, hydroxy doxorubicin; O: Oncovin®, vincristine; P: prednisone) for non-Hodgkin lymphoma, also causes mitral regurgitation and neurohumoral activation [3]. Likewise, paclitaxel and vinca alkaloids have been associated with contribution to chronic heart failure (CHF), ischemia, hypotension, atrioventricular block, ventricular tachycardia, and sinus bradycardia [4].

Accordingly, this review article focuses solely on the anthracyclines, which have been mainstay components in the treatment of many solid and hematological malignancies since the 1960s [5]. The anthracyclines comprise doxorubicin (the most commonly used formula), daunorubicin, epirubicin, and other chemical structurally related drugs, which function through a similar antitumor cytotoxic mechanism [6]. Anthracycline-induced cardiotoxicity (AIC) is dose-dependent and cumulative in both short and long term. Although the cellular damage is initially subclinical, progressive reduction in cardiac function after each administration of anthracycline can lead to increasingly serious symptoms such as CHF. Cardiotoxicity can manifest during the sessions of drug administration (acute), within a year of chemotherapy (early onset), or beyond a year of posttherapy period (late onset) [7]. Generally, cardiotoxicity can be described as various forms of cardiac dysfunction, including decrease in left ventricular ejection fraction (LVEF) with accompanying signs or symptoms of heart failure [3]. The acute effects of AIC manifest as disturbances in electrical conduction and arrhythmias. To the contrary, the early-onset and late-onset effects of AIC typically include diminished LVEF, decreased exercise capacity, and progressive symptoms of heart failure [8]. Early and late AIC are correlated with cumulative anthracycline exposure. There is a 5% risk of AIC at a cumulative dose of 400 mg/m^2^, which increases to 25% at 700 mg/m^2^ [3]. An upper limit of cumulative dosage of 500 mg/m^2^ has been suggested to reduce the development of AIC, whereas the presence of cardiovascular risk factors lowers the recommended dose to 450 mg/m^2^.

The main purpose of this review is to assess whether anthracycline chemotherapy exacerbates cardiotoxicity in patients presenting with hypertension. A thorough literature review on existing published evidence in both clinical and laboratory animal studies has been conducted with special attention paid upon the effects of a preexisting, uncontrolled, or drug-controlled hypertension on AIC in cancer patients. With the rising level of hypertension in the growing cancer patient population, understanding the effect of preexisting comorbidities on AIC will help to effectively prevent or reduce AIC among the patients with high cardiovascular risk factors. It should be emphasized that such an effort will benefit not only the population in Western developed countries, but also those in the developing countries in Africa or Latin America, where AIC has been increasingly recognized as a serious healthcare problem, as reported, for example, by investigators from Ivory Coast [9], Morocco [10], and Brazil [11].

## 2. Hypertension and Exacerbation of AIC

In 1979, von Hoff et al. first reported a possible link between hypertension and doxorubicin cardiotoxicity through a retrospective analysis of 4018 patients in the cooperative group trials [12]. In 475 patients with cardiac disease, hypertension, or both, a higher probability of developing doxorubicin-induced heart failure was observed compared to all other patients (*p* = 0.08). A retrospective study reported by Hequet et al. in 2004 in 141 lymphoma patients sought to identify the subclinical late cardiomyopathy induced by anthracyclines, five years after the successful completion of anthracycline chemotherapy for lymphoma [13]. They found that 25 (18%) of the patients had hypertension without any other heart diseases (i.e., CHF, angina, ischemic heart disease, and cardiomyopathy). Among the 25 hypertensive patients, 15 (60%) developed subclinical cardiomyopathy [13].

More recently, hypertension has been identified as the most common comorbid condition reported in cancer registries [14]. While increasing age, prior heart disease, diabetes, and hypertension all increased the risk of CHF, only hypertension intensified the effect of doxorubicin on CHF (hazard ratio = 1.8; *p* < 0.01). The patients with hypertension had a 58% higher risk of developing CHF than those without hypertension. The same study was also the first to show a significant increase in doxorubicin-related cardiotoxicity in hypertensive patients. Similarly, in older women treated with adjuvant anthracycline chemotherapy for breast cancer, hypertension was found to be a highly significant predictor of subsequent diagnosis of CHF (hazard ratio = 1.45), [15]. Another study found that 73% of the patients with diffuse large B-cell lymphoma, a type of non-Hodgkin's lymphoma, had hypertension [16]. These authors suggested accordingly that an aggressive effort to reduce hypertension in patients who are about to receive doxorubicin should be considered [16].

In a recent review of clinical predictors of AIC, while the cumulative anthracycline dosage was found to be the strongest predictor of AIC, hypertension was considered to have “an acceptable prognostic role” [30]. In 2014, Szmit et al. studied 208 lymphoma patients treated with (R)-CHOP chemotherapy (i.e., rituximab, cyclophosphamide, doxorubicin, vincristine, and prednisone) in identifying left ventricular systolic dysfunction immediately after the chemotherapy, defined by a decrease in LVEF below 50% and at least 10% from its baseline value. They found that the patients with preexisting hypertension more frequently developed decreased LVEF (19.7%) as compared with the patients without hypertension (6.6%). In turn, this caused the hypertension subgroup to have more delays in subsequent treatment (26.8% versus 14.6% in normotensive group), more reductions of doxorubicin doses (18.3% versus 8.8% in normotensive group), and early discontinuation of chemotherapy (16.9% versus 7.3% in normotensive group). This study substantiated previous evidence and further confirmed arterial hypertension as a significant risk factor predisposing to AIC [17]. Therefore it is evident that preexisting hypertension may reduce the overall effectiveness and prognosis of patients undergoing anthracycline chemotherapy.

It is notable that as old age (>65 years) has also been shown in various studies to be a strong predictor for doxorubicin-related CHF [31], many confounding factors exist in the patients with an increased age, who have higher incidence with chronic comorbidities such as diabetes or hypertension. In addition to hypertension, coronary artery disease, emphysema, diabetes, and peripheral vascular disease were also linked to higher risk of AIC.

It is noteworthy that anthracyclines are not the only class of anticancer drugs that are associated with a high risk for hypertension.  Table 1 is a comparative summary of the reported significant prohypertensive effects of several classes of oncology chemotherapeutic drugs including anthracyclines. Therefore, in addition to anthracyclines, special attention should also be paid to many other classes of anticancer drugs that are known to have high risks for drug-induced or exacerbated hypertension, especially, the angiogenesis inhibitors, including vascular endothelial growth factor (VEGF) inhibitors and tyrosine kinase inhibitors, which were discussed in detail in several recent review articles [2, 23, 25].

## 3. Cellular and Molecular Basis of AIC

The mechanism of action of AIC remains to be fully understood and is postulated to be multifactorial. A key hallmark of anthracycline-induced cardiomyocyte injury is necrotic cell death, indicated by the elevated blood levels of troponin, a specific marker of cardiac cell death, in the patients undergoing anthracycline treatment [32]. In addition, not only endothelial cells but also progenitor cells are damaged during anthracycline exposure, predisposing the heart cells to inadequate repair [3].

It has been well recognized that anthracyclines induce the generation of reactive oxygen species (ROS), through nonenzymatic or enzymatic pathways [33, 34]. Both pathways cause damage to cellular lipids, nucleic acids, and proteins, while the enzymatic pathway leads to damage, especially to the mitochondria. It was demonstrated that the levels of mitochondrial injury caused by AIC were significantly reduced in the mice with transgenic overexpression of manganese superoxide dismutase, a mitochondria-localized antioxidant enzyme [35]. Anthracycline-induced oxidative stress also activates a number of intracellular signaling pathways, involving mitogen-activated protein kinase, stress-activated protein kinase, and PI3K/Akt [36], which are critical for gene expression, cellular growth, proliferation, and survival. These protective kinase signaling pathways are often downregulated in diseased hearts, which, in turn, have diminished ability to tolerate the cumulative anthracycline exposure [37]. Other potential cardiotoxic factors of anthracyclines include the release of vasoactive amines and dysregulation of nitric oxide synthase and immune functions [38].

Furthermore, the planar structure of anthracyclines allows it to intercalate into DNA, preventing DNA and RNA synthesis, ultimately causing myocyte death. Anthracyclines are also known to poison topoisomerase, an enzyme needed for DNA replication and synthesis, further causing myocyte apoptosis [39]. Topoisomerase 2 (Top2), which exists in humans as Top2*α* and Top2*β*, is a key regulator of DNA replication, transcription, and recombination [40]. Anthracyclines function as an inhibitor of Top2*α* and Top2*β*. Top2*α* is the main target of anthracyclines, as it is required for DNA replication and is found in rapidly proliferating cells, such as tumor cells. On the other hand, Top2*β* is found in less actively dividing cells, such as cardiomyocytes, so that its inhibition leads to cardiotoxicity. Recently, Top2*β* has been described as a key mediator of AIC [41] and a potential target for cardioprotective therapy [42]. Taken together, the multifactorial effects and mechanistic complexity of AIC apparently require a multimodal approach toward its prevention and treatment.

## 4. Comorbidity of Hypertension and AIC: Potential Mechanisms

Prior damage or stressors to the heart may augment the pathophysiological effects of AIC. Nevertheless, the exact mechanisms on how the additional stress from hypertension remain largely unexplored. Hypertension is caused by a disturbance in cardiac output or systemic vascular resistance (SVR). While stroke volume is dependent on cardiac contractility and preload, SVR is dependent on vessel compliance and afterload [43]. An alteration in any of these variables can cause cardiac pathological changes. An increase in SVR could be due to a number of factors including increased alpha-adrenoceptor stimulation or increased release of angiotensin and endothelin peptides often occurred during the progression of heart failure or coronary artery disease [44]. These factors could lead to increased cytosolic calcium, causing vasoconstriction and subsequently an increase in SVR, which in turn stimulates the heart to augment its contractility in order to accommodate a higher afterload. While the heart can accommodate a higher SVR acutely, chronically, the left ventricle must undergo concentric hypertrophy to compensate such higher mechanical demands. During the cardiac remodeling, an increase in left ventricular mass and wall thickening would eventually lead to gradual decrease in contractility and filling properties, largely due to increased tissue fibrosis, and ultimately decompensated heart failure.

**Figure 1:**
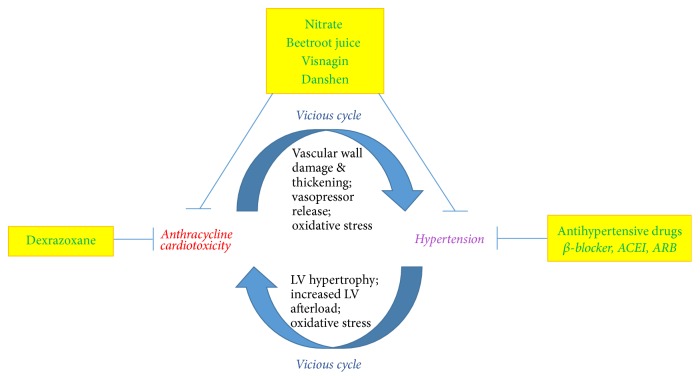
Diagrammatic summary of the vicious cycle for cardiovascular pathology under the comorbidity of anthracycline cardiotoxicity and hypertension. The current and potentially promising novel therapeutic strategies are also indicated. LV: left ventricular; ACEI: angiotensin converting enzyme inhibitor; ARB: angiotensin receptor blocker.

On the cellular and molecular levels, the pathophysiology of cardiomyocyte injuries by hypertension and AIC may be commonly related to oxidative stress and fibrotic and inflammatory processes. As mentioned above, the overproduction of ROS, beyond the capabilities of antioxidant scavengers, can lead to the damage of multiple cellular components such as DNA, membranes, and proteins [45]. Excessive ROS may impair cardiomyocyte contractility and promote cell fibrosis or death [45]. Studies have implicated the role of angiotensin II, TGF-*β*1, tumor necrosis factor *α*, members of the interleukin-6 cytokine family, growth factors, and mitogen-activated protein kinases as the mediators of myocyte damage during the transition from the compensated to decompensated heart failure [46, 47]. Although not all hypertensive patients progress to a decompensated state, a lot of the hypertension-related molecular signaling are still active in the heart [48]. In brief, anthracyclines can induce abnormal cell signaling and cytotoxic molecules that overlap with those produced by hypertension and may form a vicious cycle likely to speed up and exacerbate the process of CHF (Figure 1).

## 5. Pharmacological and Nutraceutical Protective Strategies against Hypertension and AIC Comorbidity

As anticancer treatment continues to become more and more effective, the number of cancer survivors has substantially grown during the past few decades. Therefore, it has become a clinical challenge to mitigate the resultant AIC experienced by the cancer survivors. One of the primary means of reducing AIC is by prolonging infusion and limiting the cumulative dose <450 mg/m^2^, although this limit is subject to modification depending on each individual patient [49].

### 5.1. Modifying Anthracyclines with Liposomal or Nanoparticle Preparations

Liposomal preparations were specifically designed to reduce the risk of cardiotoxicity in cancer patients, while retaining the antitumor functions of doxorubicin. In a comparison between various liposomal anthracycline formulations, including liposomal daunorubicin, nonpegylated liposomal doxorubicin, and pegylated liposomal doxorubicin, Theodoulou and Hudis found that the latter provided the strongest evidence for cardiac safety [50]. There was a significantly lower risk of cardiac events with those that were on pegylated liposomal doxorubicin. Additional studies have demonstrated that concurrent treatment with Trastuzumab potentiates the effect in women with HER-2 positive metastatic breast cancer [51]. These studies were focused on the elderly population; however, specific attention was not paid to the hypertensive comorbidity. With regard to the effect of these liposomal drug preparations on enhanced chemotherapeutic action paired with reduced cardiac events, it may be inferred that there would be a similar effect among hypertensive patients.

More recent investigations also attempted to use various nanoparticle preparations with anthracyclines that yielded encouraging results mainly in animal models with enhanced anticancer efficacy and decreased cardiotoxicity [52–54]. The effects of these doxorubicin-loaded nanoparticles on systemic blood pressure remain not well known.

### 5.2. Dexrazoxane

Dexrazoxane is a cardioprotectant that functions by chelating iron and thus reduces the formation of free radicals, which damages the myocardium [55]. It has been shown through a number of studies to offer cardioprotection during anthracycline chemotherapy for a variety of malignancies, from breast cancer to leukemia [56–58]. In 2011, the United States Food and Drug Administration released a statement restricting the use of dexrazoxane to patients with a cumulative chemotherapeutic dose of >300 mg/m^2^ of doxorubicin or >540 mg/m^2^ epirubicin. These recommendations were endorsed by the American Society of Clinical Oncology that dexrazoxane can be considered in patients with various cardiovascular risk factors, hypertension being among them [59]. In an animal model, Herman et al. showed that dexrazoxane was more cardioprotective than amifostine and prevented doxorubicin-induced mortality in hypertensive rats receiving anthracyclines treatment [60].

### 5.3. Antihypertensive Drugs

A study by Kalay et al. validated that prophylactic beta-blocker administration in patients receiving anthracycline treatment prevented the reduction of left ventricular function [61]. Similarly, administration of angiotensin-converting enzyme inhibitor reduced cardiotoxicity in high-risk cancer patients [62]. Angiotensin II receptor blockers also demonstrate decreased cardiotoxicity and nephrotoxicity. It is important to note that these drugs primarily act to reduce blood pressure, which in turn decreases the compensatory burden faced by the heart, rather than acting on the myocytes directly. By reducing the underlying hypertensive comorbidities in patients through the above prophylactic medications, significant efficacy and safety can be established in patients with anthracycline treatment.

While preexisting hypertension is an independent and critical risk factor for AIC, other anticancer drugs, such as antiangiogenic agents, are also known to cause blood pressure elevation and may exacerbate AIC when these drugs are combined in the chemotherapy regimen [63]. Therefore, it was recommended that antihypertensive intervention be administered before and after the use of these agents [64]. Carver et al. also suggested that in the patients with a high risk of cardiotoxicity the preexisting hypertensive condition should be aggressively treated [65].

### 5.4. Novel Pharmacological or Nutraceutical Therapies Targeting Both Hypertension and AIC

Dietary supplements are among the other applicable approaches to reduce AIC in cardiooncology clinics. For instance, inorganic nitrate contributes substantial health benefits in multiple organ systems, including cardiovascular [66–68], neuronal, skeletal muscle, and gastrointestinal systems [69–80]. The mechanistic basis for the benefits of beetroot juice is mainly related to dietary nitrate intake in promoting maintenance of optimal nitric oxide levels for prevention and treatment of diseases associated with nitric oxide dysregulation [81, 82]. Importantly, our preclinical animal studies in both doxorubicin cardiomyopathy [83, 84] and ischemia-reperfusion injury [85] have provided strong feasibility for conducting further clinical trials in cancer patients with both AIC and its hypertension comorbidity.

Beetroot juice is an inexpensive food product that would be ideal for long-term oral nitrate supplementation to prevent or reduce AIC [86]. Beetroot juice has been safely used in the patients with hypertension [69, 76], peripheral artery disease [80], the elderly [87], and athletes [88–90]. In fact, there are two ongoing clinical trials with beetroot juice in heart failure patients (NCT01682356 and NCT01919177). Hence, the clinical applicability of beetroot juice is promising as a robust adjunct therapy to combat against both AIC and hypertension comorbidities in cancer patients and would have a beneficial public health impact.

In addition, two other types of botanical-derived formula or compounds have been shown to possess both antihypertensive and anti-AIC properties. The first such compound is visnagin, a natural product extracted from the Eastern Mediterranean wild plant* Ammi visnaga*. The* in vivo* administration of visnagin has significant vasodilatory effect leading to a systemic hypotensive response [91]. Recently the protective effects of visnagin against AIC were also revealed and discussed [92, 93]. The other compound is Danshen, a widely used traditional Chinese herbal medicine (*Salvia miltiorrhiza*) for treating cardiovascular disorders. Tanshinone IIA was identified as the main active chemical component of Danshen, which has been shown to alleviate both hypertension [94–96] and AIC [96–99]. Taken together, these promising new therapeutic remedies (i.e., beetroot juice, visnagin, and Danshen) deserve more in-depth investigations (Figure 1), particularly, well-controlled clinical trials in cancer patients.

## 6. Conclusions

Evidence reported in the biomedical literature to date suggests that hypertension comorbidity in patients treated with anthracyclines was not thoroughly investigated and no substantial long-term data is available to mark a concrete guideline for a special therapeutic strategy to manage this predominant subgroup of cancer patients. While more evidence is needed to corroborate the additive effect of anthracycline cardiotoxicity on preexisting hypertension, this article summarizes evidence from the literature as well as animal models and supports the claim that hypertension does indeed worsen the cardiotoxic effects of anticancer chemotherapeutic drugs. In terms of management of hypertensive cancer patients, meticulous attention should be paid to pretreatment screening for risk factors, robust monitoring of cardiac function, and early intervention for preexisting comorbidities. Furthermore, several potentially effective and translatable cardioprotectants derived from botanical materials (i.e., beetroot juice, visnagin, and Danshen) should be further investigated for validating their clinical utility. Taken together, as the aging population increases and concurrently the risk of hypertension and cancer, there continues to exist the urgent need for cardiooncologists to identify novel therapeutic compound(s) and/or regimen(s), to effectively manage cancer patients with hypertension comorbidity.

## Figures and Tables

**Table 1:** Comparative summary of prohypertensive effects of anticancer chemotherapeutic drugs and the adjunvants commonly used in cancer patients.

Class of drugs	Typical drugs	Potential mechanisms for the prohypertensive effects	Representative references
Anthracyclines	Doxorubicin, daunorubicin, epirubicin, and idarubicin	Oxidative stress and apoptotic/fibrotic and inflammatory changes in vascular wall; endothelial dysfunction	[12–17]
VEGF inhibitors	Bevacizumab and vandetanib	Endothelial dysfunction; reduced nitric oxide bioavailability; increased endothelin production	[18, 19]
Tyrosine kinase inbibitors	Sunitinib, sorabenib, and pazopanib	Endothelial dysfunction; reduced nitric oxide bioavailability; vascular rarefaction; hypothyroidism	[20–22]
Alkylating agents	Cyclophosphamide and cisplatin	Endothelial dysfunction; arterial vasoconstriction; renal and vascular damage	[23, 24]
Glucocorticoids	Dexamethasone	Salt and fluid retention	[25, 26]
Erythropoietin	rhuEPO	Increase in erythrocyte mass and blood viscosity; direct vasopressor effect	[27–29]

VEGF: vascular endothelial growth factor; rhuEPO: recombinant human erythropoietin.

## Abbreviations

ACEI: Angiotensin converting enzyme inhibitor
AIC: Anthracycline-induced cardiotoxicity
ARB: Angiotensin receptor blocker
CHF: Chronic heart failure
CHOP: Cyclophosphamide, doxorubicin, vincristine, and prednisone
HER-2: Human epidermal growth factor receptor 2
LV: Left ventricle
LVEF: Left ventricular ejection fraction
PI3K: Phosphoinositide 3-kinase
(R)-CHOP: Rituximab, cyclophosphamide, doxorubicin, vincristine, and prednisone
rhuEPO: Recombinant human erythropoietin
ROS: Reactive oxygen species
SVR: Systemic vascular resistance
TGF-*β*1: Transforming growth factor *β*1
Top2: Topoisomerase 2
VEGF: Vascular endothelial growth factor.
